# Saccade reaction time asymmetries during task-switching in pursuit tracking

**DOI:** 10.1007/s00221-013-3651-9

**Published:** 2013-08-10

**Authors:** Hans-Joachim Bieg, Jean-Pierre Bresciani, Heinrich H. Bülthoff, Lewis L. Chuang

**Affiliations:** 1 Department of Human Perception, Cognition and Action, Max Planck Institute for Biological Cybernetics, Tübingen, Germany; 2 Department of Medicine, University of Fribourg, Fribourg, Switzerland; 3 Laboratoire de Psychologie et Neurocognition, CNRS, UMR 5105, Université Pierre Mendes France, Grenoble, France; 4 Department of Brain and Cognitive Engineering, Korea University, Seoul, Korea

**Keywords:** Eye movements, Saccades, Reaction time, Pursuit tracking, Smooth pursuit, Distraction, Attention, Task-switching

## Abstract

We investigate how smooth pursuit eye movements affect the latencies of task-switching saccades. Participants had to alternate their foveal vision between a continuous pursuit task in the display center and a discrete object discrimination task in the periphery. The pursuit task was either carried out by following the target with the eyes only (ocular) or by steering an on-screen cursor with a joystick (oculomanual). We measured participants’ saccadic reaction times (SRTs) when foveal vision was shifted from the pursuit task to the discrimination task and back to the pursuit task. Our results show asymmetries in SRTs depending on the movement direction of the pursuit target: SRTs were generally shorter in the direction of pursuit. Specifically, SRTs from the pursuit target were shorter when the discrimination object appeared in the motion direction. SRTs to pursuit were shorter when the pursuit target moved away from the current fixation location. This result was independent of the type of smooth pursuit behavior that was performed by participants (ocular/oculomanual). The effects are discussed in regard to asymmetries in attention and processes that suppress saccades at the onset of pursuit.

## Introduction

Saccades are discrete shifts of the eyes that place the image of an object of interest on the fovea for detailed inspection. Smooth pursuit eye movements are a different class of eye movements, which are much slower than saccades and move the eyes in a continuous fashion when following a moving stimulus (Orban de Xivry and Lefèvre 2007). The coordination of saccades and smooth pursuit eye movements is not well understood. Both types of eye movements are typically examined during fixation of a moving object. Here, smooth pursuit eye movements are complemented by saccades that automatically “catch-up” with the moving object once fixation error accumulates (e.g., de Brouwer et al. 2002b). But this is not the only class of joint ocular behavior in everyday tasks. Alternating smooth pursuit and saccadic eye movements also occur when the observer switches between tasks. For instance, drivers move their gaze back and forth between following the road and the dashboard instruments. There, saccades may also occur before or after smooth pursuit. This interaction between both classes of eye movements during task-switching has received considerably less attention.

Studies that have investigated saccade initiation during pursuit provide some insight in this regard (Tanaka et al. 1998; Kanai et al. 2003; Khan et al. 2010; Seya and Mori 2012). For example, in the study by Tanaka et al. (1998), participants pursued a moving stimulus and were instructed to switch their gaze to a second stimulus after its onset. The results show that saccade reaction times (SRTs) are asymmetric: Saccades to targets in the motion direction of pursuit exhibit shorter SRTs than saccades against the motion direction. Together with the finding that covert attention facilitates the detection of and the response to peripheral stimuli (Posner 1980, see also Deubel and Schneider 1996; Kowler et al. 1995; Hoffman and Subramaniam 1995), this phenomenon has been linked to shifts of spatial attention in the direction of pursuit in anticipation of the pursuit target’s future position (van Donkelaar and Drew 2002; Khan et al. 2010; Seya and Mori 2012; but see also Heinen et al. 2011; Lovejoy et al. 2009; Prinzmetal et al. 2005).

However, anticipatory behavior during smooth pursuit has also generally been observed in basic ocular pursuit tasks (Shagass et al. 1976; Mather and Putchat 1983; Gauthier et al. 1988; Van Gelder et al. 1990, 1995a, b; Koken and Erkelens 1992; Sweeney et al. 1994; Kathmann et al. 1999). For example, Van Gelder et al. (1990) compared pursuit performance in a standard ocular pursuit and a more naturalistic visual analysis condition. The results show a larger fixation error in the pursuit-only condition due to an increased number of anticipatory saccades that interrupted smooth pursuit. Similar results were obtained by Koken and Erkelens (1992), who showed that smooth pursuit was less frequently interrupted by saccades when it was performed during a manual tracking task rather than a basic ocular pursuit task.

The goals of the current study are twofold. First, we replicate the finding by Tanaka et al. (1998) and others (see above), namely SRT asymmetries *from* pursuit. We do this to test whether these asymmetries hinge on anticipatory behaviors that commonly occur in basic, laboratory ocular pursuit tasks (Van Gelder et al. 1995a). Second, we extend the analysis of SRT asymmetries to a more comprehensive task-switching scenario. Previous studies limited their investigation to saccades *from* pursuit and did not consider saccades *back* to the pursuit target. Such back-and-forth motion of gaze is common when a task is switched and later resumed, for instance, in our earlier example of driving. An experiment that required such task-switching was recently conducted by Jonikaitis et al. (2009). Nonetheless, this study was not primarily designed to address our current question. Thus, it did not investigate motion-related differences in saccade onsets.

To address these two questions, we used an experimental paradigm in which foveal vision was shared between a continuous pursuit task and a secondary, discrete object discrimination task. We measured the SRTs of saccades that moved the eye *away* from the pursuit target to the discrimination object and *back* to the moving pursuit target. Two variants of this task were presented. Participants followed the target either with their eyes only, which replicates the basic ocular pursuit condition that has been used by previous studies, or by steering a cursor with a joystick. In the latter condition, smooth pursuit provides important task-related information. This is expected to result in more natural eye movements, in particular, a reduction in anticipation as it can be observed in basic ocular pursuit tasks (Van Gelder et al. 1995a; Koken and Erkelens 1992).

## Methods

### Participants

Twelve participants took part in the experiment (9 males, 3 females, age 21–36 years). All participants had normal or corrected to normal vision. A vision test was conducted to verify this prior to the experiment (FrACT test Bach 2007, logMAR < 0 for all participants). In accordance with the World Medical Association’s Declaration of Helsinki, written informed consent was obtained from all subjects prior to experimentation and the procedures of the experiment had been approved by the Ethical Committee of the University of Tübingen. Participants were paid 8 EUR per hour for taking part in the experiment.

### Materials

Participants sat in an adjustable chair in front of a TFT monitor (Samsung 2233RZ, 120 Hz refresh rate, resolution 1680 × 1050, see also Wang and Nikolić 2011). A chin-rest provided support for the head at a viewing distance of 57 cm. An optical infrared head-mounted eye-tracking system was used to measure gaze at a sampling rate of 500 Hz (SR Research Eyelink II). A potentiometer joystick (0.18° angular accuracy, sampling rate 120 Hz) was mounted under the table within comfortable reach for the participants. The joystick was moved to the side of the dominant hand for each participant. With the other hand, participants pressed the cursor keys on a keyboard.

### Stimuli

Two types of stimuli were used in the experiment. Stimuli for the pursuit task consisted of differently colored vertical bars. The pursuit target was blue (RGB 180, 180, 255) and subtended 1.2° (visual angle); the cursor was orange (RGB 255, 255, 100) and subtended 0.9°. A second stimulus type was used for the object discrimination task. This stimulus consisted of a small square (0.2°) of white color (RGB 200, 200, 200). A small gap was present at one of the four sides of the square (size 0.03°, 1.8 min of arc). A white border was drawn around the target to make it discernible in the visual periphery. All stimuli were presented against a uniform gray background (RGB 100, 100, 100).

### Task

**Fig. 1:**
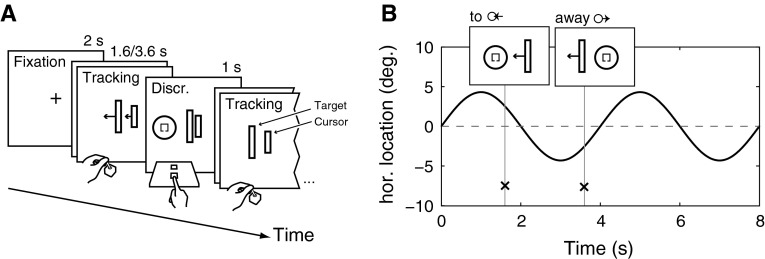
**a** Schematic of the experimental task. Each block started with an auditory warning signal and a fixation cross. This was followed by a continuous tracking block lasting 128 s. Here, participants controlled the horizontal speed of an on-screen cursor by moving the joystick to the *left* or *right*. They were instructed to follow the pursuit target as closely as possible, which moved horizontally on a sinusoidal path. The pursuit task was interrupted by a secondary task. This was an object discrimination task in which participants had to recognize the opening of a *square optotype*. **b** The pursuit target performed two full cycles every 8 s (one epoch). During each epoch, the discrimination object was presented randomly either 1.6 or 3.6 s into the epoch on the *left* or *right* side of the screen. The time and location defined whether the discrimination object was presented while the pursuit target was moving *to* the location of the discrimination object or while it was moving *away*

**Fig. 2:**
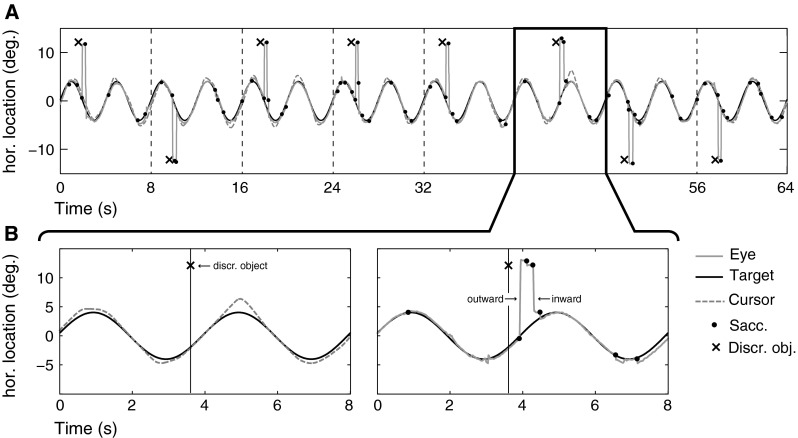
**a** Time course of stimulus presentation and response for a representative series of trials (8 trial epochs, 8 s each). **b** Close-up of one trial epoch. *Left*: sinusoidal motion of the pursuit target and time of onset (here 3.6 s into the trial, in the direction of motion) of the discrimination object. The cursor motion shows a slight overshoot when the pursuit target’s motion reaches its maximum and a pronounced overshoot after the discrimination object was presented. *Right*: the gaze movements during the same trial show periods of smooth pursuit, small catch-up saccades, and large saccades to the discrimination object (outward saccade, ca. at 4 s) and back to the pursuit target shortly afterward (inward saccade)

The primary *pursuit tracking* task required participants to steer an on-screen cursor using a joystick (see Figs. 1a, 2). By moving the joystick to the left or right, participants controlled the horizontal velocity of the cursor. The instruction was to move the cursor “as close as possible” to a computer-controlled pursuit target. The pursuit target moved horizontally in a sinusoidal path around the center of the computer screen with an amplitude of 4.3° and frequency of 0.25 Hz. This task was performed continuously in blocks, each block lasting 128 s.

The secondary *object discrimination* task required participants to look at and identify a discrimination object. Participants were instructed to discern the side of the target where the gap was located (top, bottom, left, right). Due to the small size of the gap, a saccade to the target was necessary in order to achieve this. After participants looked at the target to determine the gap, they responded with one of the four corresponding arrow keys on the keyboard.

Each pursuit block was subdivided into trial epochs of 8 s. This subdivision was not made explicitly apparent to the participants and served as a framework to control the onset of the discrimination stimulus in relation to the pursuit target motion (400 ms before the pursuit target crossed the middle of the screen). To make the repetitive appearance of the discrimination stimulus less predictable, the onset timing and location of the stimulus were varied. The stimulus appeared either 1.3 or 3.6 s after the start of an epoch at an eccentricity of 13°. The discrimination task was scheduled such that discrimination objects appeared with equal probability and frequency either in the same direction as the movement (to condition) of the pursuit target or in the opposite direction (away condition, see Fig. 1b).

Two conditions of the pursuit task were presented. In the oculomanual condition, participants controlled an on-screen cursor as described before. In the ocular condition, no cursor was present and participants were simply instructed to look at and follow the motion of the pursuit target.

### Design and procedure

The experiment was planned as a within-subjects factorial design with the factors task (ocular, oculomotor) and motion direction relative to the location of the discrimination object (to, away). The presentation of the task factor was balanced between subjects, and motion direction was varied randomly.

During a session, tasks were presented in several runs. Each run took ca. 15 min including set-up and calibration of the eye-tracker. During a run, participants performed 5 blocks of the experimental task. Regular 10-min breaks were provided after each run, during which the eye-tracker was removed. The order of task conditions was fully counter-balanced between participants, half of which began a session with ocular pursuit or oculomanual pursuit. The entire session lasted ca. 120 min.

### Data analysis

Saccade detection was carried out by the Eyelink II system using a velocity (22°/s) and acceleration threshold (3800°/ s^2^). The primary measures used to characterize saccadic eye movements were saccade reaction time (SRT), saccade amplitude, and gain. SRT was defined as the time between the onset of the discrimination object and initiation of the movement. SRTs for saccades back to the pursuit target (inward) were measured from fixation onset on the discrimination object to the beginning of a return saccade to the pursuit target. Saccade gain was defined as the size of the saccade divided by the step size, i.e., the distance between the location of gaze before the saccade and the target. For inward saccades, the pursuit target’s location at the saccade onset was used to calculate gain.

Data from the following trials were removed prior to saccade analysis: Trials with blinks during the critical time period shortly before or after the target onset, missed trials (no saccade or RT greater than 800 ms), anticipatory saccades (RT smaller than 50 ms), inaccurate saccades with errors larger 2° visual angle, and trials with blinks shortly before or after the inward saccade. Based on this method, 102 data points of 1998 were removed (5.1 %). The median number of data points remaining per participant and condition was 39 (min. 30).

For the frequency domain analysis, Fourier transforms were computed from whole 128-s blocks. Periods during which the eye moved to the discrimination object were removed by linearly interpolating between the eye position shortly before the outward and shortly after the inward saccade. The phase shift between signals was computed by subtracting the phase of the eye from the phase of the target signal at the fundamental frequency of 0.25 Hz (see also Vercher and Gauthier 1992).

If not indicated otherwise, data plots show Cousineau–Morey confidence intervals (see Baguley 2012; Morey 2008).

## Results

Separate repeated-measures ANOVAs were employed to analyze outward and inward saccades. The primary dependent variable was saccade reaction time (SRT). In addition, saccade amplitude, gain, and end point error were computed to test whether differences in SRTs could be attributed to differences in the saccade magnitude or accuracy. The primary factors under investigation were the pursuit target movement direction (to or away from the discrimination object) and the type of tracking (ocular and oculomanual). The onset time of the discrimination object during the tracking epoch was treated as a third factor since the predictability of target onsets by the observers potentially differed between both onset times (onsets occurred either early during the epoch at 1.3 s or late during the epoch at 3.6 s).

### Outward Saccades

**Fig. 3:**
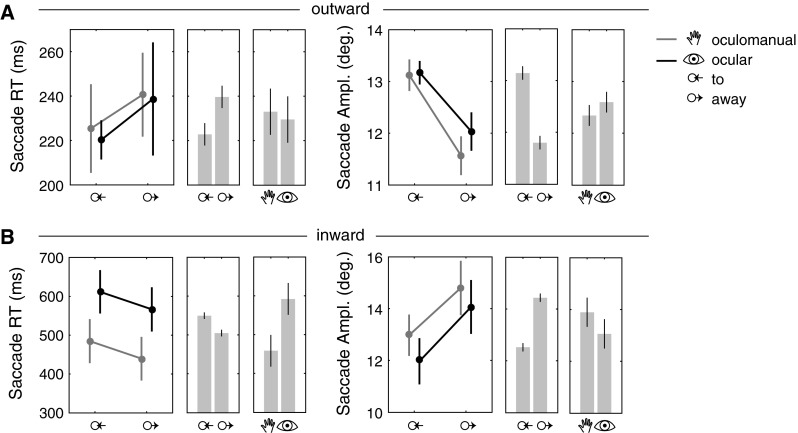
Saccade RTs and amplitudes of saccades after onset of the discrimination object (*outward*) and back to the pursuit target (*inward*) depending on task (oculomanual or ocular pursuit) and motion of the pursuit target relative to the discrimination object (to or away).* Plots* show task and motion means with standard deviations (centered on participant means).* Bar charts *show means of individual factors with 95 % confidence intervals. **a** Outward saccade RTs were shorter when the discrimination object was presented in the direction of the pursuit target’s motion. Amplitudes were larger when the pursuit target moved to the discrimination object. **b** Inward saccades were initiated earlier during the oculomanual condition and were shorter when the pursuit target moved away. Amplitudes were larger in the away condition

On average, saccades to the discrimination object (outward) were initiated after 232 ms. In both pursuit conditions, saccades that were initiated while the pursuit target moved to the discrimination object exhibited shorter RTs (222 ms) compared to saccades that started when the pursuit target moved away (240 ms,  *F*(1, 11) = 13.5, *p* < 0.01, see also Fig. 3). The analysis of the discrimination object onset times showed shorter RTs for late (225 ms) and longer RTs for early onsets (237 ms,  *F*(1, 11) = 5.9, *p* < 0.05). Interactions between all factors were not significant. This suggests that the influence of the motion direction was independent from the effect of onset time.

The eccentricity of the target at saccade onset may affect the saccade RT (Kalesnykas and Hallett 1994). To consider this possibility, we compared the amplitudes of the saccades. This showed that saccades were slightly larger when the pursuit target moved to the discrimination object (13.1°) and smaller when it moved away from the object (11.8°,  *F*(1, 11) = 128.3, *p* < 0.01).

A reduction in SRTs may be accompanied by a reduction in the accuracy of the saccades (Fischer et al. 1993). Our results show a difference in saccade gain between both motion conditions. Saccades that were initiated while the discrimination object appeared in motion direction (to condition) exhibited a lower gain (0.99) than saccades to discrimination objects at the opposite location (away condition, 1.02,  *F*(1, 11) = 6.2,  *p* < 0.05). A gain greater than one indicates overshoot, whereas a gain smaller than one indicates undershoot. To examine how this difference in saccade gain translates to fixation accuracy, we compared the magnitude of the error between the saccade end point and the discrimination target’s location. The results show no significant difference between both conditions (average absolute error 0.45°).

Depending on the SRT after onset of the discrimination object, outward saccades were either initiated before or after the eye crossed the display midline. An analysis of the starting position of outward saccades showed that saccades were initiated before the eye crossed the midline in the majority of cases (83 %). Average SRTs between both motion conditions were compared for SRTs shorter than 400 ms (before midline crossing). The main SRT results also hold for this subset: saccades that were initiated while the pursuit target moved to the discrimination object exhibited shorter RTs (210 ms) compared to saccades that started when the pursuit target moved away (238 ms,  *t*(11) = 3.5,  *p* < 0.01).

### Inward saccades

Unlike saccades to the discrimination object, saccades back to the pursuit target from the discrimination object (inward) were not triggered by an experimental signal (i.e., go signal or stimulus onset), but on participants own initiative following the discrimination task. Since we were interested in influences of the movement direction of the pursuit target on saccade performance, we first verified whether our ex-ante classification of movement direction (to/away) was valid also for inward saccades. This was necessary to test whether participants waited to saccade back after the pursuit target reached its maximal amplitude and changed direction. The analysis of inward saccade onsets showed that this was not the case. On average, and for the majority of trials (99.7 %), saccades back to the pursuit target were initiated before the pursuit signal changed its direction (825 ms after discrimination object onset on average). This means that the classification, which was based on the experimental manipulation into saccades that were initiated while the pursuit target moved to or away from the discrimination object, was correct for the majority of trials.

Saccades back to the pursuit target from the discrimination object (inward) took much longer than outward saccades (overall mean SRT 525 ms). Note that this time was measured from fixation onset on the discrimination object and therefore also comprised the discrimination time. Reaction times of inward and outward saccades can therefore not be compared directly. The ANOVA results show a statistically significant difference in SRTs for the pursuit task type (*F*(1, 11) = 13.9,  *p* < 0.01) and the motion direction of the pursuit target (*F*(1, 11) = 24.0,  *p* < 0.01). Saccades back to the pursuit target were initiated earlier during oculomanual pursuit (460 ms) compared to ocular pursuit (589 ms). SRTs were shorter when the pursuit target moved away from the discrimination object (503 ms) and longer when the pursuit target moved to the discrimination object (541 ms).

Amplitudes of inward saccades showed a significant main effect of motion direction (*F*(1, 11) = 169.0,  *p* < 0.01). Saccades were shorter when the pursuit target moved to the discrimination object (12.5°) and longer when it moved away (14.4°). The analysis of inward saccade gain showed no significant differences (average gain 0.992).

Discrimination task performance did not differ significantly between the pursuit conditions and also not between motion directions (on average 88 % correct).

### Pursuit eye movements

**Fig. 4:**
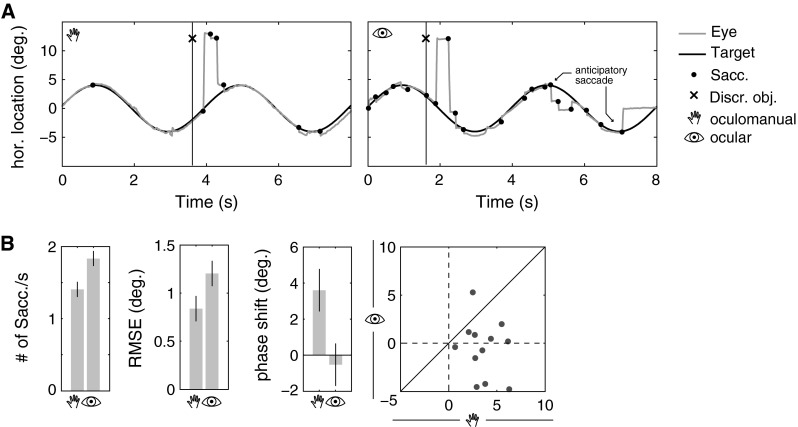
Differences in smooth pursuit behavior between ocular and oculomanual pursuit. **a** Traces of eye movements during oculomanual (*left*) and ocular pursuit (*right*) exemplify interruptions of smooth pursuit by small saccades that sometimes move gaze away from the target. **b** *Left bar charts* show average pursuit metrics with 95 % CI. The number of saccades increased and RMS error increased during ocular pursuit. On average, the eye lagged behind the target during oculomanual pursuit but preceded it slightly during ocular pursuit. *Right* the scatterplot of participant phase shift means shows that nearly half of the means were negative in the ocular condition but none was negative in the oculomanual condition

The quality of smooth pursuit eye movements was measured by counting the number of saccades per second and in the form of the RMS error and phase shift between the eye and pursuit target. Periods during which the discrimination object was present were removed prior to calculating these measures. The results show that the mean number of saccades per second was higher during ocular pursuit (1.8  saccades/s) and lower during oculomanual pursuit (1.4 saccades/s, *t*(11) = 4.5, *p* < 0.01, Fig. 4b). A larger RMS error between the eye and pursuit target was measured for ocular pursuit (1.20°) in comparison with oculomanual pursuit (0.84°,  *t*(11) = 3.1,  *p* < 0.05). The frequency spectrum of the eye movements exhibited maximal power at 0.25 Hz, which was the frequency of the target signal. The average phase shift between eye and pursuit target was lower in the ocular (−0.5°) than in the oculomanual condition (3.6°,  *t*(11) = 2.5,  *p* < 0.05). This corresponds to a lead of 5.5 ms in the ocular and a lag of 40 ms in the oculomanual condition. The phase shift of the eye was negative in 46 % of blocks in the ocular condition but only in 5 % of blocks in the oculomanual condition. Thus, in almost half of the measurements, the eye did not follow but precede the pursuit target in the ocular condition (see also Fig. 4b, phase shift). The regression of block phase shift and average number of saccades per second showed no significant correlation for the oculomanual condition (−0.004 saccades/s) but a negative slope for the ocular condition (−0.03 saccades/s, *t*(58) = 2.7,  *p* < 0.01,  *r* = 0.34). This suggests that anticipation of the target’s trajectory is accompanied by a moderate increase in saccade frequency in this condition.

## Discussion

In the current study, we examined how smooth pursuit eye movements influenced task-switching saccades. Participants alternated their gaze between a continuous pursuit and a discrete object discrimination task. The main results of our study show asymmetries in saccade reaction times (SRTs) from and to smooth pursuit depending on the smooth pursuit movement direction.

### Outward SRT asymmetry

We examined whether ongoing pursuit influenced initiation of *outward* saccades (saccades from the pursuit to the discrimination task). The SRTs of outward saccades were shorter when the saccade target appeared in the direction of the pursuit target’s movement.

In explaining this result, we first address two basic factors that are known to influence SRTs, namely the eccentricity of the saccade target and the orbital position of the eye at saccade onset. SRTs have been shown to be a function of target eccentricity (Kalesnykas and Hallett 1994, but see also Hodgson 2002; Dafoe et al. 2007). For example, in the study by Kalesnykas and Hallett (1994), longer SRTs were measured for extremely small (<2°) and very large eccentricities (>15°). Our analysis of saccade amplitudes indicates that eccentricities at the time of saccade onset differed in the two motion conditions. Saccades in the direction of motion (to condition) were larger than saccades in the opposite direction. However, considering the pattern of results of Kalesnykas and Hallett (1994), longer rather than shorter SRTs would be expected for larger eccentricities. Another factor that directly affects SRTs is the orbital position of the eye. It has been shown that centripetal saccades, saccades from an eccentric starting positions that move the eyes back to the primary position, exhibit shorter SRTs than saccades in the opposite condition (Fuller 1996; Paré and Munoz 2001). In the current experiment, orbital positions were approximately equal (namely close to the primary position) at saccade onset. This suggests that the observed SRT asymmetries are neither linked to eye nor target position but to the target’s motion direction.

This observation is in line with previous research that explains asymmetric SRTs on the basis of an attentional bias in the direction of pursuit (Tanaka et al. 1998; Kanai et al. 2003; Khan et al. 2010). For example, Khan et al. (2010) showed shorter SRTs to targets ahead of the pursuit stimulus and longer SRTs to targets behind the pursuit target. This phenomenon has also been reported for manual response times in a detection task (van Donkelaar 1999; van Donkelaar and Drew 2002). This suggests that SRT asymmetries are not due to biomechanical compatibility between saccade and pursuit direction but rather an example for an attention shift in the direction of pursuit. Khan et al. (2010) suggest that orienting of covert attention in anticipation of the pursuit target’s motion is important such that potentially required actions can be planned ahead, in compensation for neural processing delays in perception and action.

The results of our study exclude a basic explanation for this attentional bias. In pursuit tasks that do not require a visual analysis of the target of some sort, the pursuit behavior is not completely smooth but shows discontinuities in the form of anticipatory saccades (Van Gelder et al. 1990, see also Koken and Erkelens 1992; Xia and Barnes 1999). According to Van Gelder et al. (1990, 1995a, b; Kathmann et al. 1999), pursuit is typically performed automatically to support the visual analysis of the target. Without such a visual function, attention is unnaturally focused on pursuit itself, which may explain any anticipatory behavior. Our hypothesis was that this tendency to anticipate the pursuit target’s motion could have also caused the attentional bias and reduction in SRTs in the motion direction. However, the current results speak against this assumption. SRTs were asymmetric in both pursuit conditions (ocular, oculomanual), despite clear differences in smooth pursuit behavior in regard to anticipation (see also Mather and Putchat 1983; Gauthier et al. 1988; Vercher and Gauthier 1992; Koken and Erkelens 1992). This suggests that pursuit-related attentional biasing is not merely the result of confined experimental settings and extends its relevance to more realistic conditions.

### Inward SRT asymmetry

We tested whether SRT asymmetries also existed for *inward* saccades (saccades from the discrimination object to the pursuit target). SRTs were shorter when the saccade target moved away from the current fixation location (foveofugal) and longer when it moved toward the fixation location (foveopetal).

Like outward saccades, SRTs were shorter when the saccade moved the eye in the same direction as the pursuit target. An advantage for saccades that are compatible with the pursuit motion direction was explained for saccades *from* pursuit by a broad attentional bias in the direction of pursuit, which facilitates detection and processing of targets that appear in this direction (Blohm et al. 2005; Khan et al. 2010). An alternative explanation suggests that it is not a sustained bias in attention but facilitation of attention capture, which leads to reduced SRTs to sudden target onsets in the direction of pursuit (Lovejoy et al. 2009).

Neither theory sufficiently explains the current results. First, when fixating on the discrimination object prior to the inward saccade, pursuit targets were situated in the same visual hemifield at similar visual field locations in both motion conditions. A broad tuning of attention would therefore be expected to affect saccades in both conditions. Second, inward saccades were not triggered by a sudden target onset. Instead, saccades followed the discrimination task and moved the eye to the pursuit target, which was continuously present throughout the experiment. Hence, facilitation of attention capture is also unlikely to explain the obtained result.

In the remainder of this discussion, we will consider several alternative explanations, namely the influence of the discrimination task, amplitude differences, motion processing asymmetries, compatibility with early pursuit responses, and inhibition of saccades at the onset of smooth pursuit.

The amount of time spent on performing the discrimination task may explain differences in SRTs. For example, longer discrimination times may be the result of inaccurate foveation after the outward saccade. However, our analysis provides no evidence for this assumption. Discrimination performance and saccade accuracies were similar in both conditions. In addition, the current finding is corroborated by data from a different experiment, in which neither discrimination nor a saccade was required before the saccade to pursuit (Bieg et al. 2013, in preparation).

Factors that influence SRTs more directly are the eccentricity of the saccade target and the orbital position of the eye at saccade onset. However, the eccentricity differences in our experiment would predict the opposite effects on SRTs (see previous section). This suggests that the observed SRTs are primarily influenced by the motion direction of the pursuit target.

Asymmetries in the processing of motion have been observed in several experiments. But the conditions that would lead to an advantage in one or the other direction (foveofugal/foveopetal) are not clear (Naito et al. 2010). For example, in an experiment by Ball and Sekuler (1980), RTs to motion onsets of foveofugal motion were shorter. Other experiments showed an advantage for foveopetal motion (Mateeff and Hohnsbein 1988; Mateeff et al. 1991a, b; Raymond 1994; Jancke et al. 2004). One reason for these conflicting findings could be differences in the presented type of motion. Mateeff et al. (1991b) compared flow-field motion (i.e., random-dot kinematograms) stimuli and single-target motion stimuli. The latter stimulus is similar to the one that was used in the present experiment. Mateeff et al. (1991b) show that stimuli of this sort lead to processing advantages of foveopetal motion (in terms of onset detection) rather than foveofugal motion, as in our experiment (in terms of SRTs). These findings speak against an explanation on the basis of motion processing asymmetries.

Potentially related to asymmetries in motion processing are asymmetries in smooth pursuit behavior. These can be observed during the early (ca. 100 ms), open-loop pursuit response (Tychsen and Lisberger 1986; Carl and Gellman 1987). This response can occur at the onset of pursuit and moves the eyes in the direction of the pursuit target’s motion. Investigations of this response showed larger early accelerations during foveopetal motion (Tychsen and Lisberger 1986). This initial acceleration could potentially affect saccade onsets by modulating the omnipause neuron activity in the brain stem. Inhibition of these neurons is required to trigger a saccade (Scudder et al. 2002), and they also likely regulate smooth pursuit onset and gain (Missal and Keller 2002; Kornylo et al. 2003; Krauzlis 2005). Inhibition of omnipause activity due to early pursuit responses could therefore facilitate saccade triggering. With regard to the findings by Tychsen and Lisberger (1986), stronger inhibition of omnipause neurons would be expected when the target moves foveopetally, which would explain shorter SRTs in this direction. Again, this is incompatible with the results that we observed, namely shorter SRTs to foveofugal motion.

Apart from this hypothetical facilitatory connection, pursuit-related activity is known to *inhibit* saccades in certain conditions. Increased SRTs or even complete suppression of a saccade can be observed in foveopetal step-ramp tasks. There, the target is stepped in the opposite movement direction such that it moves across its original position after a specified time. This time is the *zero-crossing* or *eye crossing* time (Gellman and Carl 1991; de Brouwer et al. 2002a). In the case of zero-crossing times of 200 ms, the initial saccade to the target position is delayed or suppressed completely and smooth pursuit of the target commences directly (Rashbass 1961; Gellman and Carl 1991). It is currently unknown how this cancelation process affects saccades for zero-crossing times larger than 200 ms. For example, the study by Moschner et al. (1999) measured SRTs in step-ramps with 200 ms zero-crossing times. Their results show longer SRTs in foveopetal steps (ca. 400 ms) and shorter SRTs in foveofugal steps (ca. 200 ms). However, this difference in SRTs can be primarily attributed to cancelation of the initial saccade and re-planning of a new saccade in the direction of motion after zero-crossing. In contrast, SRT differences in inward saccades in our experiment cannot be attributed to cancelation and re-planning since (1) zero-crossing never actually occurred and (2) hypothetical zero-crossing times were much longer: An estimate based on the average amplitude prior to saccade onset (12.5°) divided by the pursuit target speed (max. 6.7°/s, average before onset 5.2°/s) results in zero-crossing times between 1.8 and 2.4 s.

It cannot be excluded that the same mechanisms that lead to cancelation of saccades in short zero-crossing times also influence saccade generation in longer zero-crossing times. Saccade triggering as well as cancelation are thought to depend on neuronal accumulation processes (Carpenter and Williams 1995; Hanes and Schall 1996). Importantly, there is also evidence for inhibitory links between those processes (Boucher et al. 2007). Assuming that cancelation of saccades to foveopetal motion is indeed organized by such a process network, foveopetal motion would be expected to have a stronger impact on the cancelation process gain than foveofugal motion. The inhibitory connections between the two processes can then explain increased SRTs to foveopetal motion. In this respect, it is important to point out that an asymmetry in SRTs may also be behaviorally useful. In foveofugally moving targets, computation of the exact time of zero-crossing from a motion analysis of the pursuit target becomes obsolete. Considering that a more precise motion estimate also requires more time (Bruyn and Orban 1988; Bennett et al. 2007), it would be efficient to allocate less time for the analysis of foveofugal rather than foveopetal motion, in particular because foveofugal motion moves the target out of the visual field, which poses the danger of losing track of it entirely when the saccade is triggered too late. In this respect, the ensuing reduction in saccade RTs may additionally be related to time pressure (Reddi and Carpenter 2000; Montagnini and Chelazzi 2005; Bieg et al. 2012).

## Conclusion

We examined how smooth pursuit eye movements influenced initiation of saccades in the context of task-switching. Here, gaze had to be switched from and to a pursuit task. First, our results confirm earlier findings, which show that the relative movement direction of the pursuit stimulus affects saccade reaction times (SRTs) *from* pursuit. Our results also provide evidence against a potential explanation for this behavior, namely the tendency to anticipate the pursuit target’s trajectory, which is particularly pronounced in basic, laboratory pursuit tasks (Van Gelder et al. 1995a).

Second, our results show that saccades *to* pursuit are similarly affected by the relative movement direction of the pursuit target. We speculate that the difference in SRTs may be caused by the processes that organize cancelation of saccades at the onset of pursuit movements (Rashbass 1961; Gellman and Carl 1991). Additional studies are required to establish the exact conditions, for example, changes in the zero-crossing time (de Brouwer et al. 2002b), that lead to these SRT differences. This would allow a more precise specification to which extent saccades are influenced when switching to pursuit behavior.
